# Prediction of protein-protein interaction sites using an ensemble method

**DOI:** 10.1186/1471-2105-10-426

**Published:** 2009-12-16

**Authors:** Lei Deng, Jihong Guan, Qiwen Dong, Shuigeng Zhou

**Affiliations:** 1 Department of Computer Science and Technology, Tongji University, Shanghai 201804, China; 2 Shanghai Key Lab of Intelligent Information Processing, Fudan University, Shanghai 200433, China; 3 School of Computer Science, Fudan University, Shanghai 200433, China

## Abstract

**Background:**

Prediction of protein-protein interaction sites is one of the most challenging and intriguing problems in the field of computational biology. Although much progress has been achieved by using various machine learning methods and a variety of available features, the problem is still far from being solved.

**Results:**

In this paper, an ensemble method is proposed, which combines bootstrap resampling technique, SVM-based fusion classifiers and weighted voting strategy, to overcome the imbalanced problem and effectively utilize a wide variety of features. We evaluate the ensemble classifier using a dataset extracted from 99 polypeptide chains with 10-fold cross validation, and get a AUC score of 0.86, with a sensitivity of 0.76 and a specificity of 0.78, which are better than that of the existing methods. To improve the usefulness of the proposed method, two special ensemble classifiers are designed to handle the cases of missing homologues and structural information respectively, and the performance is still encouraging. The robustness of the ensemble method is also evaluated by effectively classifying interaction sites from surface residues as well as from all residues in proteins. Moreover, we demonstrate the applicability of the proposed method to identify interaction sites from the non-structural proteins (NS) of the influenza A virus, which may be utilized as potential drug target sites.

**Conclusion:**

Our experimental results show that the ensemble classifiers are quite effective in predicting protein interaction sites. The Sub-EnClassifiers with resampling technique can alleviate the imbalanced problem and the combination of Sub-EnClassifiers with a wide variety of feature groups can significantly improve prediction performance.

## Background

Protein-protein interactions are critical to nearly all aspects of cellular function, such as regulation of metabolic and signaling pathways, immunological recognition, DNA replication and gene translation, as well as protein synthesis [1]. In particular, identifying the binding sites between two interacting proteins provides important clues to the function of a protein and the structural elucidation of protein complexes, thus helps identifying pharmacological targets and guides drug design. Hence, solving the puzzle of predicting the interaction sites is of great significance to molecular recognition.

Many of the existing studies focus on the identification of protein-protein interaction sites with specific physicochemical and geometric characteristics. Binding sites have been widely observed to be more hydrophobic, planar, globular and protruding than outer surfaces [2-6]. Different amino acid compositions have also been found among the interaction sites of homo-permanent complexes, homo-transient complexes, hetero-permanent complexes, and hetero-transient complexes [7]. Interfaces have a significant number of polar residues [8,9], where usually the interactions are less permanent [10]. Through alanine-scanning mutagenesis, it has been observed that the binding free energy is not distributed equally across these protein interfaces. Residues of interface, protein core, and non-interface surface are found significantly different in sequence entropy and secondary structure [11]. However, secondary structure composition appears to be of little discriminatory power, because neither *α*-helices nor *β*-sheets dominate at transient binding sites [12]. Furthermore, evolutionary profiles and conservation score have been used in locating binding sites [13-15] with some success, since the interface core tends to be more conserved than the periphery in both obligate and non-obligate cases [16].

Based on different kinds of characteristics, several machine learning approaches have been proposed for predicting protein-protein interaction sites, such as neural networks [15,17-19], support vector machines [13,14,20-24], Bayesian network [25], hidden Markov models [26] and conditional random fields [27]. For these methods, a local neighborhood or a window is used as input, to predict protein-protein interface residues at a particular amino acid sequence, with a single characteristic or a combination of features, such as hydrophobic distribution, residue composition, sequence profile, evolutionary conservation, accessible surface area, structural conservation score, and so on.

Although much progress has been made, the problem of predicting interaction sites is still far from being solved. There are several reasons for this difficulty. Firstly, specific biological properties for precisely identifying protein-protein interaction sites are not fully exploited [28], no single parameter can absolutely differentiate interfaces from other surface patches [22,29]. For example, hydrophobicity is an average characteristic of interacting surfaces in homodimers, but has only limited power of predicting interaction sites in some types of complexes. A number of studies have attempted to combine more than one of these characteristics discussed above. Secondly, the existing techniques, which use conventional orthogonal encoding or information derived directly from the amino acid sequences as input to predict the protein-protein interaction residues, are similar in performing string analysis on protein sequences [23]. Thirdly and also most importantly, the imbalanced problem exists widely in protein interaction site prediction because the number of interacting sites of a protein is usually much smaller than that of non-interacting sites [28]. The imbalanced data tends to cause over-fitting and poor performance, in particular on the interacting class. To solve the imbalanced problem, a series of solutions have been proposed at both algorithmic and data levels, including one-class learning algorithm, feature selection, and resampling technique.

Recently, Zhao et al. [30] proposed a new algorithm with a hybrid sampling technique and a committee of classifiers, which have been successfully applied to protein homology detection. Chen and Jeong [28] developed a random forest-based integrative model, which consists of multiple decision tree predictors with randomly selected variable subsets. Improved performance was achieved in comparison with two other sequence-based methods [13,31] by aggregating the predictors. In this study, inspired by the methods used by Zhao and Chen, we propose a hybrid approach, which incorporates bootstrap resampling technique, SVM-based fusion classifiers and weighted voting strategy, to overcome the imbalanced problem and consequently improve the performance of protein interaction sites prediction. Also, a wide variety of features are extracted from amino acid sequences and structures. They are grouped into four categories and transformed by two methods. Therefore, a total of eight different feature spaces are obtained to further improve the performance of the hybrid approach. The experiments, using a 10-fold cross validation procedure on 99 polypeptide chains, produce promising results and validate the effectiveness of the proposed approach.

## Methods

### Datasets

The datasets used in this study are quite similar to those in the works by Chen and Jeong [28]. Firstly, the individual proteins are extracted from a set of 70 protein-protein heterocomplexes used in the study of Chakrabarti and Janin [32]. Proteins with sequence identity less than 30% are subsequently obtained after removing redundant proteins and molecules with less than 10 residues. Some proteins that are not available in HSSP [33] and DSSP [34] programs are also omitted. As a result, 99 polypeptide chains are extracted from 54 heterocomplexes, which can be grouped into six categories according to the scheme of Chakrabarti and Janin [32]. The categories and the number of representatives in each category (the values in the parentheses) are as follows: antibody antigen (29), protease-inhibitor (19), enzyme complexes (14), large protease complexes (8), G-proteins (13) and miscellaneous (16). The surface residues are defined based on their relative solvent accessible surface area (RASA), which is calculated by the DSSP program [34]. A residue is considered as a surface residue if its RASA is greater than 25%. A total of 13,771 surface residues are collected from all these polypeptide chains. Furthermore, a surface residue is defined to be an interface residue if its calculated ASA in the complex (CASA) is less than that in the monomer (MASA) by at least 1*Å*^2 ^[8]. By this way, the number of protein-protein interaction sites is about 10% (2,828 residues) of the whole set of residues contained in the selected polypeptide chains (27,442 residues). Therefore a total of 2,828 interaction sites are obtained as positive samples and 24614 non-interface residues are defined as negative samples (Additional file 1: Dataset).

### Evaluation measures

The performance of the proposed ensemble method is measured using 10-fold cross-validation. Each data set is randomly divided into ten subsets with an approximately equal number of polypeptide chains. Each classifier is trained and tested ten times with one dataset. And for each time, nine subsets are used as training data and the remaining subset is used as test data.

Some widely used measures in information retrieval research are adopted in this study, such as *sensitivity *(recall), *specificity*, *correlation coefficient *(CC) and *AUC *(area under ROC curve) score. These measures are defined as follows:

where the *TP*, *FP*, *TN *and *FN *are abbreviations of the number of true positive, the number of false positive, the number of true negative and the number of false negative, respectively. The *AUC *score is the normalized area under the ROC curve. The ROC curve is plotted with true positives as a function of false positives for various classification thresholds.

### Feature extraction

In our experiment, a wide variety of characteristics are selected for the protein interaction sites classification, including physicochemical features, evolutionary conservation score, information entropy, position-specific scoring matrices (PSSMs), solvent accessible area (ASA), normalized atom contacts (*NC*_*a*_) and normalized residue contacts (*NC*_*r*_).

#### Physicochemical features

The six physicochemical properties of amino acids are hydrophobicity, hydrophilicity, polarity, polarizability, propensities and average accessible surface area (Additional file 2: Physicochemical features). The original values of the six physicochemical properties for each amino acid are obtained from the AAindex database [35].

#### Evolutionary conservation score

Evolutionary conservation score is based on multiple sequence alignments (MSAs) and phylogenetic tree. Following the method used by ConSurf [36], amino acid sequences similar to each other in the PDB [37] are collected by using PSI-BLAST [38] and then multiple aligned by using MUSCLE [39]. The evolutionary conservation of each amino acid position in the alignment is calculated by using the Rate4Site algorithm [40].

#### Sequence entropy

Sequence entropy values for residues are extracted from the HSSP database [33]. They are normalized over the range of 0-100, with the lowest sequence entropy values corresponding to the most conserved positions [11].

#### Position-specific scoring matrices (PSSMs)

PSSMs are taken from multiple sequence alignment obtained by PSI-BLAST [38] searching against NCBI non-redundant database ftp://ftp.ncbi.nih.gov/blast/db/, with parameters *j *= 3 and *e *= 0.001.

#### Solvent accessible area (ASA)

ASA features represent the relative accessible surface areas, which are calculated by using DSSP program [34] for each residue in the unbound state.

#### Normalized atom contacts (*NC*_*a*_)

The normalized atom contacts (*NC*_*a*_) of a residue (e.g. the *i*-th residue) are computed by summing all atom contacts (*C*_*a*_) between the residue and any other amino acid (e.g. the *j*-th residue) in the sequence, then dividing the sum by the number of atoms in the residue (*N*_*a*_(*i*)), as described by Equation (1). Contact between two atoms (*C*_*a*_) is defined in Equation (2). This equation shows that if two atoms are located within a cutoff distance of 5.0*Å *[41-43], then they are in contact. Atoms contained in neighboring residues are not considered to be in contact.(1)(2)

#### Normalized residue contacts (*NC*_*r*_)

Similarly, the normalized residue contacts (*NC*_*r*_) of the target residue are calculated by summing all residue contacts of the residue and dividing the sum by the number of atoms in the residue, as represented in Equation (3). The contact between two residues is defined in Equation (4), which indicates that if one residue contact exists, at least one atom contact exists between the two residues.(3)(4)

Based on previous studies and our experimental validation, the above characteristics are combined into four groups so as to get better performance.

• Group 1 - physicochemical features, evolutionary conservation score and sequence entropy.

• Group 2 - position-specific scoring matrices (PSSMs), evolutionary conservation score and information entropy.

• Group 3 - position-specific scoring matrices (PSSMs), solvent accessible area (ASA), normalized atom contacts (*NC*_*a*_) and normalized residue contacts (*NC*_*r*_).

• Group 4 - physicochemical features, solvent accessible area (ASA), normalized atom contacts (*NC*_*a*_) and normalized residue contacts (*NC*_*r*_).

Thus, we can extract 8, 22, 23, 9 features for group 1, group 2, group 3 and group 4, respectively. No structure-based feature is contained in the first two feature groups, while there is no evolutionary information included in the last feature group. In order to build predictors for interaction site classification, each polypeptide chain with these features needs to be converted into a fixed length feature vector. Most of the existing techniques use the conventional orthogonal encoding for this transformation. In this paper, we utilize both *conventional orthogonal encoding *and *auto covariance *(AC) transformation for each feature group. The conventional orthogonal encoding uses input vector of 21 contiguous amino acid residues, corresponding to a sliding window containing the target residue and 10 neighboring residues on either side of the target residue. Each of the 21 residues in the window is represented by 8-bit, 22-bit, 23-bit and 9-bit vector for the four feature groups respectively.

Auto cross covariance transformation (ACC) is a new feature representation, which has been adopted by more and more investigators for protein classification [44,45]. ACC results in two kinds of variables, AC between the same descriptor, and cross covariance (CC) between two different descriptors. For each residue sequence, AC variables describe the average interactions between residues, in a certain range of *d *throughout the whole sequence, as represented in Equation (5). Here *j *represents one descriptor, *j *= 1,2, ⋯, *D *(*D *is the number of descriptors); *i *denotes the position in the sequence; *L *is the length of the amino acid sequence and *lg *is the maximum of *d *(*d *= 1,2, ⋯, *lg*). The number of AC variables for each sequence can be calculated as lg*D.(5)

In this paper, we use only the AC variables to transform the numerical vectors of 21 contiguous amino acid residues into uniform matrices, with parameter *lg *= 10. Since the total number of CC variables is about *D *- 1 times as many as that of AC variables, there will be thousands of dimensions in feature space after ACC transformation. If a combination of AC and CC variables is adopted, although a little better performance may be obtained, it would be rather costly in running time and unsuitable especially for an ensemble classifier. More details about auto cross covariance transformation can be seen in our previous work [46].

With the four feature groups and the two transform methods described above, we can obtain eight different feature spaces.

### Sub-Ensemble classifiers

**Figure 1:**
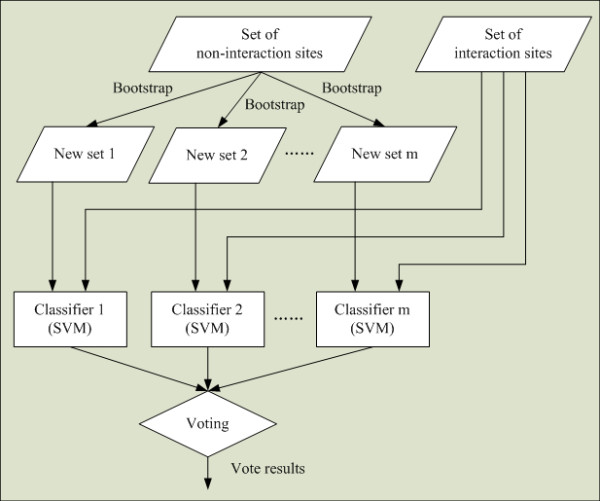
**Overview of the sub-ensemble classifier**. The number of non-interaction sites is much more than that of interaction sites.

In this section, we first present a component ensemble classifier, namely *Sub-EnClassifier*, to effectively utilize every feature space and to handle the imbalanced classification problem. Figure 1 shows the overview of the proposed component ensemble classifier. As in most cases, the number of non-interaction sites (majority class) is much more than that of interaction sites (minority class), and the ratio of sizes between them is usually larger than three. To deal with the imbalanced problem, the Sub-EnClassifier uses an ensemble of *m *classifiers and decision fusion technique on the training set of each feature space. An asymmetric bootstrap resampling approach [47,48] is adopted to generate subsets for all component classifiers. It performs random sampling with replacement only on the majority class so that its size is equal to the number of minority samples, and keeps the entire minority samples in all subsets.

In the first step, the majority class of non-interaction sites is under-sampled and split into *m *groups by random sampling with replacement, where each group has the same or similar size as the minority class of interaction sites. After the sampling procedure, we obtain *m *new datasets from the set of non-interaction sites. Each of the new dataset and the set of interaction sites are combined into *m *new training sets. Then, we train *m *classifiers by using the *m *new training sets as inputs, with one classifier corresponding to one training set. Each of these classifiers is a Support Vector Machine (SVM). Here the LIBSVM package 2.8 http://www.csie.ntu.edu.tw/~cjlin/libsvm/ is used with radial basis function as the kernel. Finally, a simple majority voting method is adopted in the fusion unit, and the final result is determined by majority votes among the outputs of the *m *classifiers for further processing with 10-fold cross-validation.

### Combination of Sub-Ensemble classifiers of different feature spaces with weighted voting

**Figure 2:**
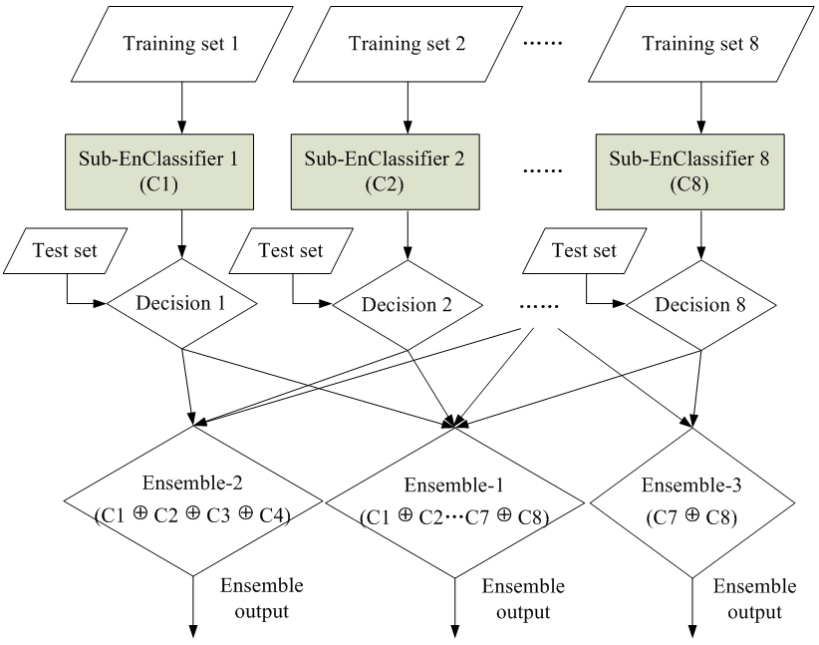
**Schematic diagram of ensemble classifiers**. The Ensemble-1 utilizes all features while Ensemble-2 and Ensemble-3 use the first four and the last two feature spaces respectively.

Since there are eight different feature spaces established by the four feature groups and the two transform methods, for each feature space, we generate a Sub-EnClassifier. Figure 2 is the schematic diagram for an ensemble classifier that combines eight Sub-EnClassifiers. The final output of the ensemble classifier is the weighted fusion of the outputs produced by the eight individual Sub-EnClassifiers. Suppose the ensemble classifier ℂ (called *Ensemble-1*) is expressed in Equation (6), where ℂ1, ℂ2, ⋯, ℂ8 represent the eight Sub-EnClassifiers, the symbol ⊕ denotes the fusing operator.(6)

Concretely, the process that the ensemble classifier ℂ (Ensemble-1) works by fusing the eight Sub-EnClassifiers ℂ*i *(*i *= 1,2, ⋯, 8) can be formulated as follows:(7)

where *w*_*j *_is the weight of the *j*-th Sub-EnClassifier, *P*_*j *_indicates the prediction made by the *j*-th Sub-EnClassifier.

In order to get proper weight parameters (*w*_*j*_) that would result in a classifier with good predicting performance, a restricted grid search method is introduced in this work. Comparing with many other optimization algorithms, the grid search may find a global, rather than a local, optimum, but it might be rather costly in computation time. We develop a restricted grid search strategy to select the values of these eight weight parameters. The principles of this strategy are: (1) the weight of any Sub-EnClassifier can only take the values from 0 to 1, with a step of 0.05; and (2) the sum of the weights of all eight Sub-EnClassifiers should be equal to 1. With the two restrictions, the search space for the eight parameters is effectively limited to a reasonable size. To evaluate the performance of the ensemble classifier, a two-level 10-fold cross-validation is conducted. For each round in the first level, nine folds of examples are utilized to train the Sub-Ensemble classifiers and the remaining fold is used as test set, based on the test results of which, a second level 10-fold cross-validation is implemented, where nine folds of the test results are used to select optimal parameters for weighted voting and the remaining test results are used to test.

In some occasions, the evolutionary information cannot be obtained if no homologue of the query protein is found. On the other hand, the structure information is often unavailable for many proteins since the number of proteins with known structures is much smaller than that of proteins with known sequences. To handle the problem of incomplete or missing data, two special ensemble classifiers are designed. The sequence-based classifier (called *Ensemble-2*) uses the first four feature spaces that contain no structure-based feature, formally, ℂ1⊕ℂ2⊕ℂ3⊕ℂ4, and the *Ensemble-3 *utilizes the last two feature spaces without evolutionary information, that is, ℂ7⊕ℂ8. These two ensemble classifiers are trained in the same way as Ensemble-1.

## Results and Discussion

### Evaluation of different feature combinations

In previous studies, many combinations of features have been adopted to get improved predictions of protein interaction sites, these combinations include: sequence profile and evolutionary rate [13]; position-specific scoring matrix (PSSM) and accessible surface area (ASA) [23]; sequence profile, ASA and evolutionary conservation score [27]; sequence profile, ASA and structural conservation score [21]; physicochemical features, evolutionary conservation score, amino acid distance and PSSM [28]. Based on these studies, we construct a variety of component ensemble classifiers (Sub-EnClassifiers) to investigate the performance of different feature combinations, the detailed results are depicted in Table 1. It can be seen that classifiers with combined features outperform the classifiers based on component attributes alone. When the physicochemical features are combined with evolutionary conservation score and sequence entropy, there is at least 5% increase in AUC score, 4% increase in sensitivity and specificity. The combination of PSSM, evolutionary conservation score and sequence entropy outperforms the combination of PSSM and evolutionary conservation score, with a 2% improvement on AUC score, which means that sequence entropy is helpful to performance enhancement. When the feature ASA is combined with normalized atom contacts (NCa) and normalized residue contacts (NCr), the improvement on performance is impressive, at least 3% increase in AUC score and sensitivity, which implies that the novel features NCa and NCr play an important role in performance improvement. Among these feature groups, the combination of PSSM, ASA, NCa and NCr obtains the best performance, with a AUC score more than 0.84. The combination of physicochemical features, ASA, NCa and NCr also gains a relatively high performance. These enhancements on performance indicate that the features contained in the combinations may be complementary, and that exploiting this complementarity is helpful for predicting interaction sites.

**Table 1:** The results of the classifiers with different features and feature combinations.

Features	AUC	Sensitivity	Specificity
Phy	0.67536	0.58547	0.66764
PSSM	0.75496	0.68039	0.68796
ASA	0.74781	0.76540	0.65852

Phy+ECS+Entropy*	0.72944	0.62285	0.70575
PSSM+ECS	0.75812	0.69484	0.67211
PSSM+ECS+Entropy*	0.77802	0.69670	0.71086
ASA+NCa+NCr	0.77408	0.79943	0.65921
PSSM+ASA+NCa+NCr*	0.84647	0.76836	0.76798
Phy+ASA+NCa+NCr*	0.83079	0.73978	0.75002

Phy means physicochemical features while ECS is the abbreviation of evolutionary conservation score. The symbol '*' denotes the selected feature group.

### Performance of Sub-EnClassifiers

**Figure 3:**
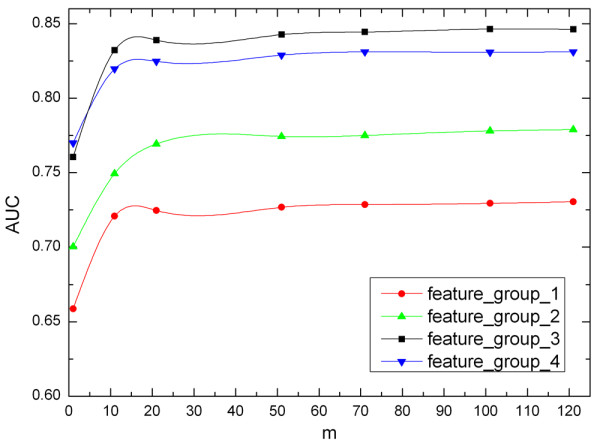
**Performance vs. the value of *m***. The curves illustrate AUC scores obtained from Sub-EnClassifiers for four feature groups, with different values of *m*.

Before applying the proposed method to predict protein interaction sites, the value of parameter *m *in Figure 1 needs to be determined. The parameter *m *represents the number of negative examples (non-interaction sites) partitions and the number of classifiers in the Sub-EnClassifier to be trained. In this study, four Sub-EnClassifiers are constructed for each feature group with different values of *m*. Figure 3 shows the impact of parameter *m *on the AUC score. A remarkable improvement can be found on AUC score when *m *increases from 1 to 20. However, after *m *is larger than 20, its impact on performance is slight. In order to maximize the use of negative examples and restrict the computational cost to a reasonable level, a value of 100 is chosen for the parameter *m *in this paper after carefully tuning.

To evaluate the performance of the proposed Sub-EnClassifiers, a comparison experiment is conducted by using imbalanced data, randomly trimmed data and our re-sampled data. Such comparison experiment has been widely conducted in previous studies. The imbalanced data includes the complete examples in the original datasets, and the the randomly trimmed data is generated by selecting negative examples randomly with a 1:1 ratio of positive to negative examples. The imbalanced data and the trimmed data are used directly for SVM classifier training. It is worth pointing out that all of the SVM classifiers use the same parameters. Table 2 shows the experimental results, where the first column denotes the inputs of four feature groups. It can be seen from Table 2 that the results on the balanced data are better than that on imbalanced data and trimmed data for any feature group. The results also show that imbalanced data can lead to higher specificity but lower sensitivity. The comparison results confirm that Sub-EnClassifiers with the proposed re-sampling technique can effectively deal with imbalanced data and obviously improve prediction performance.

**Table 2:** The results over imbalanced, trimmed and balanced data.

Features	Dataset	AUC	Sensitivity	Specificity
Group 1	Imbalanced	0.71815	0.23618	0.95589
	Trimmed	0.69259	0.60103	0.67216
	Balanced	0.72944	0.62285	0.70575

Group 2	Imbalanced	0.74139	0.26960	0.96100
	Trimmed	0.74575	0.73217	0.62533
	Balanced	0.77802	0.69670	0.71086

Group 3	Imbalanced	0.81745	0.37526	0.95015
	Trimmed	0.81670	0.72653	0.74426
	Balanced	0.84647	0.76836	0.76798

Group 4	Imbalanced	0.80099	0.27180	0.96929
	Trimmed	0.79362	0.72478	0.71542
	Balanced	0.83079	0.73978	0.75002

The imbalanced data includes all examples in the original datasets; the trimmed data owns all positive examples and randomly selected negative examples, with a 1:1 ratio of positive to negative examples; the balanced datasets are generated by Sub-EnClassifiers with resampling technique.

### Parameter selection with restricted grid search

Based on each fold of the first level cross-validation results from Sub-EnClassifiers, a restricted grid search method is conducted to select optimal weight parameters for weighted voting with a second level 10-fold cross-validation. It uses AUC score as weighting scheme to assess the performance of different parameter combinations. For each time in the second level cross-validation, the test results from Sub-EnClassifiers are divided into ten folds, nine folds of which are utilized to select optimal parameters that maximize the AUC score, and the remaining fold is used as test set. The parameters of Ensemble-1 selected in each round are shown in Table 3, Sub-EnClassifiers with higher predicting performance seem to get relatively higher weight.

**Table 3:** Optimal weight parameters of Ensemble-1 selected by the restricted grid search on each round of 10-fold cross-validation.

Round	*w*_1_	*w*_2_	*w*_3_	*w*_4_	*w*_5_	*w*_6_	*w*_7_	*w*_8_
1	0.0	0.0	0.2	0.1	0.0	0.4	0.05	0.25
2	0.05	0.0	0.1	0.1	0.0	0.35	0.0	0.4
3	0.05	0.15	0.0	0.1	0.0	0.35	0.0	0.35
4	0.0	0.0	0.0	0.05	0.0	0.35	0.05	0.55
5	0.0	0.0	0.05	0.0	0.0	0.6	0.0	0.35
6	0.05	0.15	0.0	0.05	0.2	0.35	0.0	0.2
7	0.05	0.0	0.05	0.3	0.1	0.4	0.0	0.1
8	0.0	0.0	0.0	0.0	0.0	0.4	0.05	0.55
9	0.0	0.0	0.05	0.0	0.0	0.75	0.1	0.1
10	0.1	0.1	0.0	0.0	0.15	0.4	0.0	0.25

A total of 100 groups of parameters are obtained in the two-level 10-fold cross-validation. Since the parameters selected in the second level cross-validation are similar to that of each round, only a representative group of parameters for each second level cross-validation is listed here.

### Performance of ensemble classifiers with weighted voting

**Figure 4:**
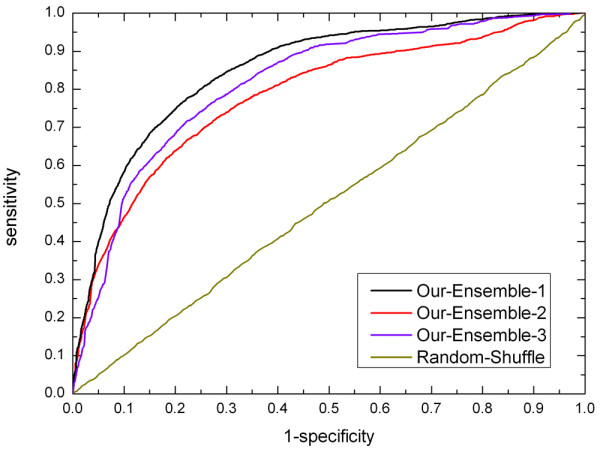
**ROC curves of ensemble classifiers with different inputs**. The four curves are obtained based on Ensemble-1, Ensemble-2, Ensemble-3 and randomly shuffled test, respectively. The Ensemble-1 classifier uses all the features, while Ensemble-2 uses only sequence features without structure information and Ensemble-3 uses the last two feature spaces without evolutionary information.

In this section, the performance of ensemble classifiers with weighted voting is investigated. Figure 4 shows the ROC curves of the ensemble classifiers with different inputs. The ROC curves are constructed by changing the threshold we put on the results of weighted voting. The classifier Ensemble-1 uses all of the eight feature spaces, while the sequence-based Ensemble-2 uses the first four feature spaces without structure-based features and Ensemble-3 uses the last two feature spaces without evolutionary information. The random shuffle test uses randomly labeled sets that are generated by randomly shuffling the class labels for all the examples. From Figure 4 we can see that the areas under the four curves are about 0.86, 0.79, 0.83 and 0.49 respectively. This clearly indicates that the three ensemble classifiers are all significantly better than a random predictor. Also, the performance of Ensemble-1 is better than that of Ensemble-2 and Ensemble-3. For example, with a specificity rate of 0.75, the sensitivities of Ensemble-1, Ensemble-2, Ensemble-3 and random shuffle test are 0.80, 0.69, 0.74 and 0.25, respectively.

**Figure 5:**
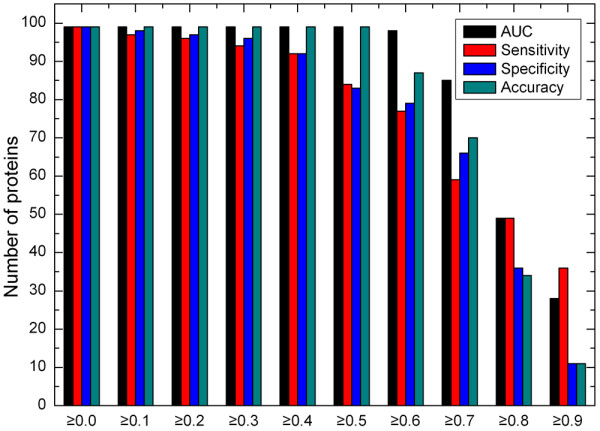
**Interaction site prediction: the distribution of the number of proteins against different performance measure thresholds for 99 polypeptide chains**. The horizontal axis stands for thresholds of different performance measures, including AUC, sensitivity, specificity and accuracy; the vertical axis means the number of proteins in the prediction results satisfying different performance thresholds, a larger value corresponds to better prediction performance.

Figure 5 shows the distribution of the number of proteins against different performance measures of Ensemble-1 for 99 polypeptide chains. This experiment is based on the complete dataset, with which eight Sub-EnClassifiers are constructed for weighted voting. In Figure 5, The horizontal axis stands for thresholds of different performance measures, including AUC, sensitivity, specificity and accuracy; the vertical axis means the number of proteins in the prediction results satisfying different performance thresholds, a larger value corresponds to better prediction performance. It can be seen that the AUC scores are greater than 0.7 for over 85% of the proteins. The distributions against sensitivity and specificity values indicate that there is a quite good balance of prediction accuracy between positive examples and negative examples. When the measure threshold exceeds 0.6, there are at least 75% of proteins for sensitivity, while more than 78% of proteins for specificity. Furthermore, at least 60% residues are correctly classified for over 85% of the proteins.

In order to compare the effectiveness of the ensemble classifiers with that of component Sub-EnClassifiers, we list in Table 4 the performance of these classifiers. For the AUC score, Ensemble-1 with complete features outperforms all component Sub-EnClassifiers, while Ensemble-2 and Ensemble-3 are also more effective than the first four sequence-based Sub-EnClassifiers. Some component Sub-EnClassifiers have lower sensitivities though their specificities are relatively higher, which indicates that these classifiers have more false negatives. The results in Table 4 also verify the effectiveness of the ensemble strategy with weighted voting.

**Table 4:** Performance comparison: ensemble classifiers vs. Sub-EnClassifiers

Methods	AUC	Sensitivity	Specificity
Sub-EnClassifier1	0.65289	0.45974	0.73808
Sub-EnClassifier2	0.72944	0.62285	0.70575
Sub-EnClassifier3	0.70759	0.46654	0.81004
Sub-EnClassifier4	0.77802	0.69670	0.71086
Sub-EnClassifier5	0.70238	0.47541	0.79535
Sub-EnClassifier6	0.84647	0.76836	0.76798
Sub-EnClassifier7	0.64221	0.39094	0.76555
Sub-EnClassifier8	0.83079	0.73978	0.75002

Ensemble-1	0.86273	0.76334	0.78611
Ensemble-2	0.79189	0.74009	0.70019
Ensemble-3	0.83117	0.73637	0.75139

### Comparison with other classification methods

Since the datasets utilized in our experiments are quite similar to those in the work by Chen and Jeong [28], it is reasonable to compare our ensemble classifier with Chen and Jeong's method directly. As described in Chen and Jeong's paper, with a specificity of 0.7, they achieved a sensitivity of 0.73 and a CC value of 0.28, while our ensemble method with complete features (Ensemble-1) obtains a sensitivity of 0.83 and a CC value of 0.37, and our sequence-based classifier (Ensemble-2) reaches a sensitivity of 0.74 and a CC value of 0.29. Comparing with the random forest-based model, both our ensemble classifier with complete features and our sequence-based classifier achieves better prediction performance. It is worth emphasizing that the number of features used in our sequence-based classifier is much smaller than that in Chen and Jeong's work.

**Figure 6:**
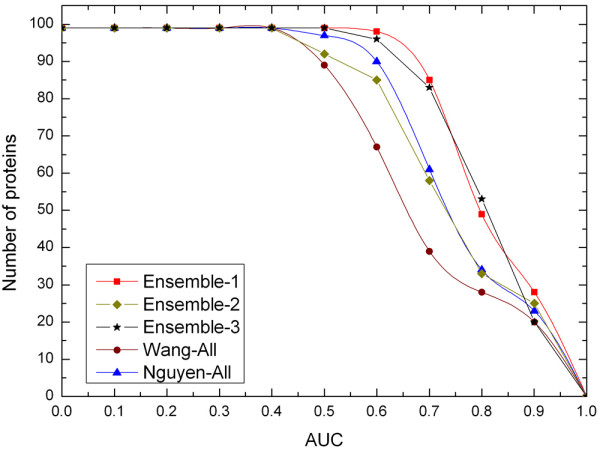
**Performance comparison of our method with Wang's and Nguyen's**. The graph plots the total number of proteins whose AUC scores are higher than specific threshold. Each curve corresponds to one prediction method.

Moreover, we compare our proposed classifiers with other two methods. The first method was proposed by Wang et al. [13], which uses PSSM and evolutionary conservation score with 11 neighbor residues, the second was introduced by Nguyen et al. [23], which uses PSSM and accessible surface areas (ASA) with 15 neighbor residues. Both of the two methods use SVM to construct classifiers. For the sake of making a fair performance comparison, we implement Wang' and Nguyen's methods to classify the same datasets of ours with 10-fold cross-validation. The results are shown in Table 5, which indicate that our ensemble classifier (Ensemble-1) produces the best AUC score of 0.86, compared to Wang's 0.72 and Nguyen's 0.80. The classifier Esemble-3 without using evolutionary features also outperforms Nguyen's and Wang's methods considerably. For the sequence-based ensemble classifier Ensemble-2, its performance is nearly similar to that of Nguyen's method but better than that of Wang's method. In addition, prediction results of Nguyen's are also observed a lower sensitivity and a higher specificity, which indicates that positive examples are predicted much worse than negative examples, probably because of the imbalanced datasets used in the experiments. Furthermore, the distribution of protein number versus AUC score threshold for the four methods are plotted in Figure 6, where a higher curve corresponds to more accurate performance. We can also see that the classifier Ensemble-1 and Ensemble-3 outperform all the other methods and the performance of the sequence-base ensemble classifier (Ensemble-2) is comparable with that of Nguyen's method.

**Table 5:** Performance comparison: our method vs. Wang's and Nguyen's methods

Methods	AUC	Sensitivity	Specificity	CC
Wang-All	0.72867	0.69760	0.66619	0.23023
Nguyen-All	0.80262	0.43635	0.92561	0.34937

Ensemble-1	0.86273	0.76334	0.78611	0.37627
Ensemble-2	0.79189	0.74009	0.70019	0.29368
Ensemble-3	0.83117	0.73637	0.75139	0.32403

Although considerable performance improvement is observed by our classifiers, one problem is: do the ensemble classifiers or the new combinations of features contributes to the performance improvement? and which contributes more? To investigate this problem, we generate two classifiers with our component Sub-EnClassifiers based on the features of Wang's and Nguyen's. The AUC scores of the two classifiers are 0.75, 0.82 respectively, which outperform the results of Wang's and Nguyen's methods based on the same features by a rate of 3% and 2%. On the other hand, the ensemble classifiers with our four new combinations of features, achieve a AUC score of 0.86. These results imply that both the component Sub-EnClassifiers and the new combinations of features play important role in performance improvement, and the new combinations of features seem to make a little more contribution.

In previous studies, some researchers predicted interaction sites only from surface residues rather than the ways used in our experiments, which directly classify all residues (including surface and non-surface residues) into interaction residues and non-interaction residues. To further evaluate the robustness of the proposed ensemble classifiers, three additional experiments are implemented to predict interaction sites from surface residues by utilizing the methods of Wang's, Nguyen's and ours (Ensemble-1). The results of the three experiments are reported in Table 6. It clearly shows that the performance of our ensemble method outperforms the other two methods to a great extent, especially the sensitivity value. As is well known, a higher sensitivity means a better prediction in positive classes, and is very useful for correct identification of interface residues. Though Nguyen's method obtained a higher specificity, its sensitivity is very low, such a predictor is useless in practical applications. For our proposed ensemble classifiers, predicting interaction residues from surface residues is as effective as that from all residues.

**Table 6:** Performance comparison: our method vs. Wang's and Nguyen's methods.

Methods	AUC	Sensitivity	Specificity	CC
Wang-All	0.71933	0.68640	0.65417	0.28026
Nguyen-All	0.74943	0.35980	0.92949	0.33247
Our-Ensemble	0.79761	0.76765	0.63158	0.34562

Predicting interaction sites from surface residues, when non-surface residues are removed from negative examples.

**Figure 7:**
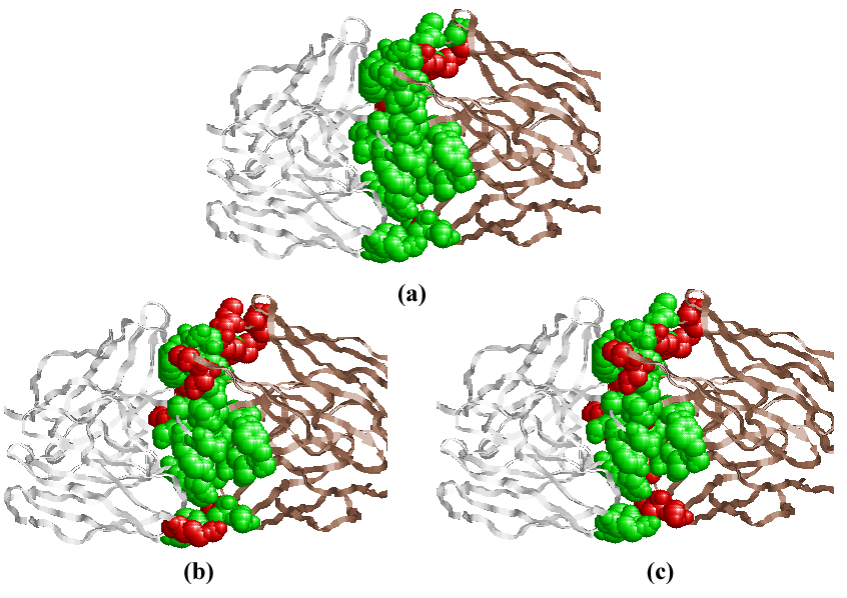
**The visualization of prediction results of chains **1IAI**:LH and **1IAI**:MI in **1IAI. The residues are obtained by using (a) our method, (b)Wang's method and (c) Nguyen's method. Green sphere denotes true positives while red sphere indicates false negatives.

Finally, a test on protein complex 1IAI (PDB code) is conducted as an example to further illustrate the effectiveness of our approach by using the RasMol software [49]. The prediction results are shown in Figure 7, the green sphere denotes true positives (true interaction residues that are correctly predicted) and the red sphere indicates false negatives (true interaction residues that are predicted as non-interaction residues). Both green spheres and red spheres denote all the interaction sites in 1IAI. We can see that most interaction residues can be predicted correctly by our ensemble method, and our method can identify more interaction residues than the other two methods.

### Evaluation with the independent test dataset

An independent test is constructed to further validate the usability of our ensemble method (Ensemble-1). We train the classifiers based on the dataset of interaction residues and non-interaction residues from the 99 proteins with the proposed ensemble methods and the same parameters as before. Then we test our constructed classifiers against the dataset of Bradford and Westhead [22]. The test dataset contains 180 proteins taken from 149 complexes, with sequence identity <20% and the number of residues >20. The results of the test are very encouraging, with an AUC score of 0.81, a sensitivity of 0.72 and a specificity of 0.75. More than 74% of the residues are successfully predicted. The CC value in the test is 0.35, while a CC value of 0 corresponds to random guessing.

### Locating potential drug targets

Since many diseases are caused by abnormal protein-protein interactions, locating potential sites of these interactions on a protein surface is critical to designing inhibitor drugs. Here, we concentrate on demonstrating by our method how to predict protein-protein interaction sites involved in the non-structural protein of influenza A virus, which causes the current world-wide flu pandemic. The natural host of influenza A viruses is waterfowl, however, influenza A viruses also infect humans and other animals such as pigs, horses and various avian species [50]. Influenza pandemics seems to occur when a pathogenic avian type virus acquires the capability of efficient human to human transmission [51], which may occur due to mutations or reassortment of human and avian RNA segments [52]. The non-structural proteins (NS, including NS1 and NS2) of influenza A viruses play important role in the infectious life cycle of the virus. NS1 is a non-essential virulence factor that has multiple accessory functions during viral infection. The major role ascribed to NS1 has been its inhibition of host immune responses, especially the limitation of both interferon (IFN) production and the antiviral effects of IFN-induced proteins [53]. It is clear that NS1 also acts directly to modulate other important aspects of the virus replication cycle. The NS2 protein is referred to as nuclear export protein (NEP) according to its role in mediating the export of viral ribonucleoproteins from the nucleus to the cytoplasm through nuclear export signals and independent interaction with human chromosome region maintenance protein Crm1 [54]. It is potentially involved in viral assembly through its interaction with the M1 protein that plays a key role in virus assembly [55].

**Figure 8:**
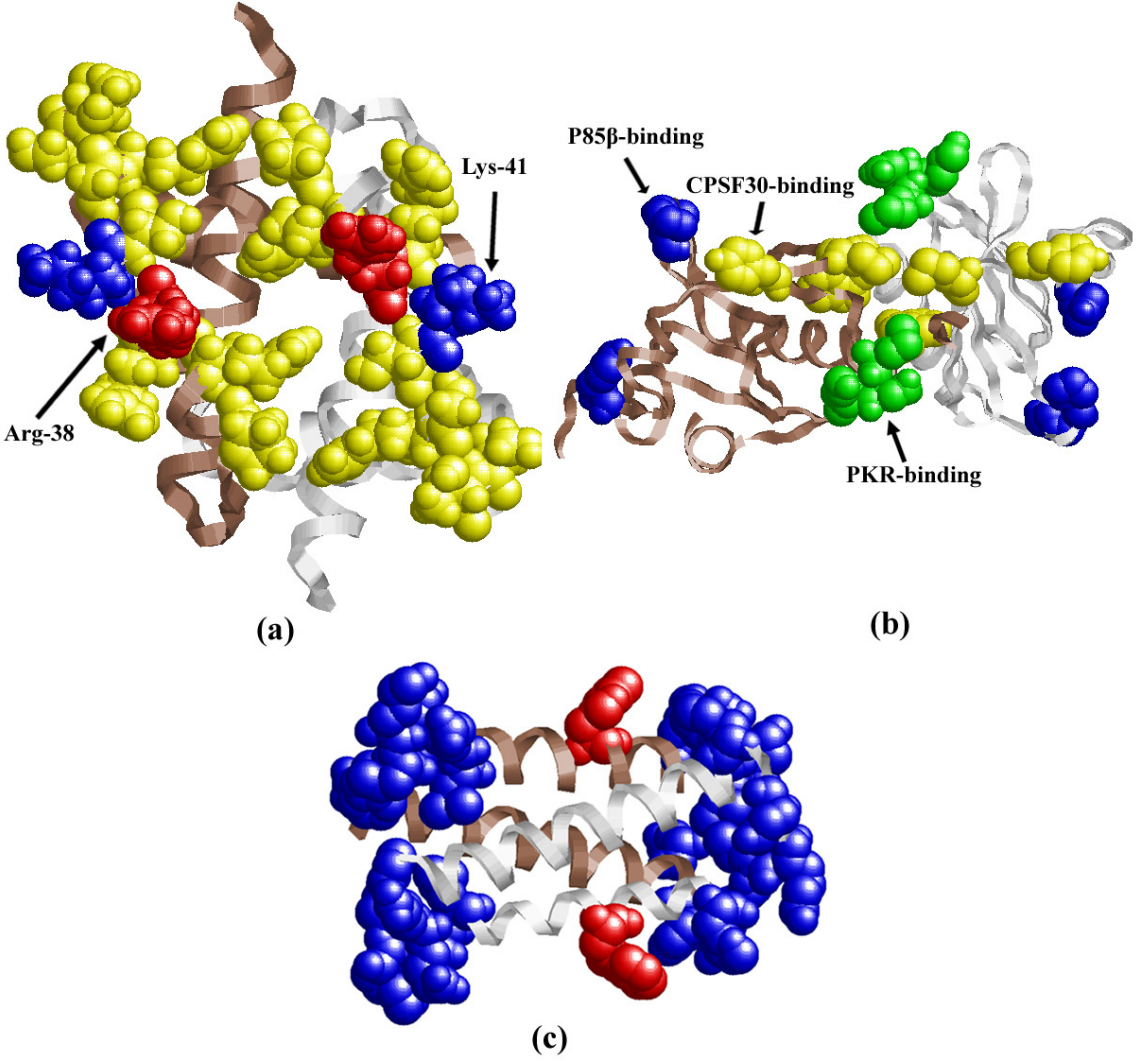
**Prediction results of the influenza A virus NS proteins using our method**. For all structures (a-c), monomers are colored goldenrod and brown. The figures are generated by using RasMol. (a) Structure of 1NS1, red and blue spheres are correctly predicted atoms of Arg-38 and Lys-41, yellow spheres denotes other predicted atoms. (b) Structure of 3D6R, yellow, green and blue spheres denote correctly predicted residues in binding CPSF30, PKR and p85β, respectively. (c) Structure of 1PD3, red spheres denote correctly predicted Trp-78, blue spheres denotes other predicted residues.

NS1 is notionally divided into two distinct functional domains: an N-terminal RNA-binding domain and a C-terminal effector domain [56]. We first investigate the structure of the RNA-binding domain (PDB ID: 1NS1), which is a symmetrical homodimer with each monomer consisting of three *α*-helices. Two identical helices from each NS1 monomer contribute towards dsRNA-binding by forming antiparallel 'tracks' on either side of a deep cleft [57]. As shown in Figure 8a, Arg-38 and Lys-41, which are critical to RNA-binding, are correctly predicted. Our method also predicts some other residues (colored yellow), including Pro-31, Arg-35, Arg-37, Ser-42, Gly-45, Arg-46, Ser-48 and Thr-49, some of which may be investigated as potential drug target sites. Then, we test the structure of 3D6R, which is the effector domain of avian influenza virus A/Duck/Albany/76 (see Figure 8b). Many of the predicted residues are implicated in binding CPSF30 (the 30-kDa subunit of cleavage and polyadenylation specificity factor [58], colored yellow), P85*β *(a regulatory subunit of phosphatidylinositol-3-kinase [59], colored blue) and PKR (the dsRNA-dependent serine/threonine protein kinase R [60], colored green), which are in agreement with previous studies.

For the NS2 protein, the structure of the M1 protein-binding domain (PDB ID: 1PD3) [61] reveals an amphipathic helical hairpin, which dimerizes as a four-helix bundle. The predicted results are shown in Figure 8c. The red spheres denote the predicted Trp-78, which is surrounded by a cluster of glutamate residues and considered to be highly conserved [61]. A variety of other binding sites (colored blue) are predicted, including residues 64-69, 84-88, Glu-91 and Phe-94. These predicted residues are very close to the binding sites observed in the study of Darapaneni et al. [62]. Residues of Arg84, Leu87, Lys88 and Glu91 are located at the protein surface in an apical position and contain four conserved residues, of which Arg84 is highly conserved. This may define a previously unknown interaction site, which could be investigated as a potential drug target site. At the opposite apex, residues 64-67 are located and contain two highly conserved residues, Trp65 and Arg66.

## Conclusions

In this paper, we have shown a novel ensemble method using bootstrap resampling technique to handle the imbalanced problem and SVM-based fusion classifiers to increase the accuracy of classification on protein-protein interaction sites. The novelty of our approach also lies in the way we combine the selected features and the weighted voting strategy for fusing the results of component element predictors (Sub-EnClassifiers). We evaluate the ensemble classifiers and compare them with several other existing methods on the dataset of 99 polypeptide chains with 10-fold cross validation. The results clearly show that the suggested ensemble classifiers are quite effective in predicting protein binding sites. Our classifier achieves a satisfactory AUC rate of 0.86, which is significantly better than that of the compared methods. The experiment results also show that the Sub-EnClassifiers with resampling technique can alleviate the imbalanced problem and the combination of Sub-EnClassifiers with a wide variety of features can significantly improve prediction accuracy. The robustness of the ensemble method is evaluated by classifying interaction sites from both surface residues and all residues in proteins effectively. Moreover, our classifiers can work well in the occasions of missing homologues and structure information, and achieves good AUC scores of 0.83 and 0.79 respectively. Finally, we demonstrate the applicability of our method to drug discovery process by successfully predicting a number of interaction sites in the NS proteins of influenza A viruses. These predicted sites may be utilized as potential drug target sites for developing universal anti-influenza drugs.

For the future work, more effective features and transform methods will be investigated. Other machine learning algorithms such as neural networks, k-NN, decision trees and logistic regression will also be considered in the ensemble classifiers. In addition, the proposed ensemble method can also be applied to other protein classification problems.

## Authors' contributions

LD carried out the literature study, developed the new ensemble method and drafted the manuscript. JG participated in its design and coordination. QD and SZ helped to draft and revise the manuscript. All authors read and approved the final manuscript.

## Supplementary Material

Additional file 1 **Dataset used in our experiments**. The dataset contains 2828 interface sites and 24614 non-interface residues. Click here for file

Additional file 2 **Physicochemical features**. Values of the six physicochemical features are contained in this file. Click here for file
